# Prevalence of *Plasmodium* spp. in humans and cattle: Implications for zoonotic malaria transmission in Indonesia

**DOI:** 10.14202/vetworld.2025.1831-1839

**Published:** 2025-07-08

**Authors:** Hamzah Hasyim, Babucarr Jassey, Ririh Yudhastuti, Misnaniarti Misnaniarti, Iche Andriyani Liberty, Elvi Sunarsih, Langgeng Priyanto, Dalilah Dalilah, Yusri Yusri, Fildzah Hashifah Taufiq, Fadhilah Eka Maharani, Lukman Hakim, Siti Herlinda

**Affiliations:** 1 Department of Public Health, Faculty of Public Health, Universitas Sriwijaya, 30662 Indralaya, Indonesia; 2 Department of Public Health, Faculty of Public Health, Universitas Airlangga, Surabaya, East Java, Indonesia; 3 Department of Public Health Services, Ministry of Health, Quadrangle Banjul, The Gambia; 4 Department of Animal Science, Faculty of Agriculture, Universitas Sriwijaya, 30662 Indralaya, Indonesia; 5 Department of Parasitology, Faculty of Medicine, Universitas Sriwijaya, 30662 Indralaya, Indonesia; 6 Department of Biology, Faculty of Mathematics and Natural Science, Bogor Agricultural University, 1668 Bogor, Indonesia; 7 Department of Malaria, Ministry of Health, Republic of Indonesia, 12950 Jakarta, Indonesia; 8 Department of Plant Protection, Faculty of Agriculture, Universitas Sriwijaya, 30662 Indralaya, Indonesia; 9 Research Center for sub-optimal Lands (PUR-PLSO), Universitas Sriwijaya, 30662 Indralaya, Indonesia

**Keywords:** cattle, Indonesia, malaria, One Health, *Plasmodium* spp, zoonoses

## Abstract

**Background and Aim:**

Zoonotic malaria remains a significant public health concern in Southeast Asia. The potential role of cattle as reservoirs for *Plasmodium* spp. in Indonesia has not been fully elucidated, despite increasing recognition of animal reservoirs in malaria transmission dynamics. This study aimed to investigate the prevalence of *Plasmodium* spp. in humans and cattle in a malaria-endemic region of Indonesia to explore the potential for zoonotic transmission and inform integrated control strategies aligned with Sustainable Development Goal 3.3.

**Materials and Methods:**

A cross-sectional study was conducted between March to July 2024 involving 41 human participants and 43 cattle. Blood samples were collected and analyzed using endpoint polymerase chain reaction techniques targeting *Plasmodium* genus-specific DNA sequences. The infection prevalence in both populations was determined, and the results were interpreted to assess the risk of zoonotic malaria transmission.

**Results:**

All human blood samples tested negative for *Plasmodium* spp., corresponding to a 0% infection rate (95% confidence interval [CI]: 0.0%–8.5%). In contrast, one cattle sample tested positive, resulting in a 2.33% infection rate among cattle (95% CI: 0.06%–12.0%). The positive detection in cattle was confirmed by a distinct 240 base pairs band through agarose gel electrophoresis. The absence of infections in humans suggests the effectiveness of current public health measures, while the presence of *Plasmodium* DNA in cattle underscores the potential role of cattle as parasite reservoirs.

**Conclusion:**

The findings highlight the importance of integrating animal health surveillance into malaria elimination programs under the One Health framework. Although no zoonotic transmission to humans was observed, the detection of *Plasmodium* spp. in cattle warrants continuous surveillance, improved livestock management practices, and targeted vector control measures. Further studies with species-specific molecular diagnostics and broader geographic coverage are recommended to clarify the zoonotic potential and transmission dynamics involving cattle.

## INTRODUCTION

Parasitic zoonoses continue to present a major global public health challenge, particularly in Southeast Asia, where factors such as deforestation, agricultural expansion, and close interactions between humans and cattle significantly influence disease transmi-ssion dynamics [1]. Malaria, caused by *Plasmodium* species, remains a major public health concern in Indonesia, creating considerable obstacles to control and elimination efforts. The intricate interactions among parasites, vectors, and both human and animal hosts further complicate the transmission of malaria. Environmental changes, including deforestation and agricultural development, coupled with close human-cattle associations, are recognized as major contributors to malaria prevalence and distribution in the region [1, 2].

Recent research by Alasil and Abdullah [3] has highlighted the emergence and re-emergence of parasitic infectious diseases of significant public health relevance in neighboring countries such as Malaysia, largely attributed to increased migration from endemic areas and limited public health awareness among at-risk populations. These socioeconomic dynamics are similarly relevant to Indonesia, where rural communities frequently live in close proximity to livestock, potentially promoting zoonotic transmission cycles. The role of cattle in malaria transmission remains complex and under active investigation. While some studies propose that cattle may serve a protective role by diverting mosquito vectors away from humans – a phenomenon known as zooprophylaxis – other studies suggest that cattle may act as reservoirs for *Plasmodium* parasites [4].

This study is theoretically anchored in the eco-health framework, which conceptualizes diseases within the broader context of social-ecological systems, emphasizing the interconnectedness of human, animal, and environmental health [5]. Understanding the dynamics of the animal–vector–human interface is crucial for developing effective strategies to control malaria. The rise of zoonotic malaria caused by simian *Plasmodium* species, such as *Plasmodium knowlesi*, in Southeast Asia further underscores the need for vigilant monitoring of *Plasmodium* spp. in both human and animal populations [1]. Such zoonotic pathogens have been identified as major contributors to malaria incidence in Malaysia, raising concerns about potential parallels in Indonesia [3].

Despite the acknowledged significance of cattle in malaria epidemiology, limited data exist on the prevalence of *Plasmodium* spp. in cattle populations within Indonesia, and the implications for human health remain poorly understood. A prior study by Hasyim *et al*. [3] has largely concentrated on human malaria cases or vector surveillance, often neglecting the role of cattle as potential reservoirs. For instance, Hasyim *et al*. [4] reported an increased malaria risk associated with indoor cattle husbandry but did not examine the actual prevalence of *Plasmodium* spp. in cattle themselves. Similarly, Kaltsum *et al*. [6] detected *Plasmodium* spp. parasites in cattle in Muara Enim Regency, highlighting the need for further investigation. This knowledge gap hinders the formulation of comprehensive control strategies that consider all possible transmission routes. Furthermore, the detection of *Plasmodium falciparum* and *Plasmodium vivax* in domestic animals in highly endemic regions such as Papua suggests that human *Plasmodium* parasites may be transmitted not only through mosquitoes but also through vertebrate reservoir hosts [7].

Although zoonotic malaria has emerged as a critical concern in Southeast Asia, particularly due to the rise of *P. knowlesi* infections in Malaysia, the role of domestic animals, particularly cattle, in malaria transmission cycles in Indonesia remains inadequately understood. Previous studies have primarily focused on human malaria cases and vector ecology, with limited attention given to livestock as potential reservoirs. Notably, while Hasyim *et al*. [4] identified an increased malaria risk associated with cattle-rearing practices, the direct detection of *Plasmodium* spp. in cattle populations was not assessed. Furthermore, isolated findings, such as the detection of *Plasmodium* spp. in cattle by Kaltsum *et al*. [6] in Muara Enim Regency, suggest the presence of animal reservoirs; however, these studies have not been comprehensive or systematically correlated with human infection data within the same regions. In addition, while *P. falciparum* and *P. vivax* have been reported in domestic animals in other endemic areas like Papua; there remains a lack of integrated investigations simultaneously evaluating humans and cattle within Indonesian malaria-endemic settings. This gap impedes the development of One Health-based malaria control strategies that incorporate surveillance of animal reservoirs alongside human and vector monitoring. The absence of species-specific identification of *Plasmodium* detected in cattle further limits the understanding of zoonotic potential and epidemiological significance.

This study aimed to investigate the prevalence of *Plasmodium* spp. in humans and cattle within a malaria-endemic region of Indonesia through molecular diagnostic approaches, specifically endpoint polymerase chain reaction (PCR). By simultaneously analyzing blood samples from human participants and cattle residing in the same communities, this research sought to elucidate the potential role of cattle as reservoirs for *Plasmodium* parasites and assess the implications for zoonotic transmission dynamics. The findings aim to address critical knowledge gaps regarding non-human reservoirs in malaria transmission and inform the development of integrated, One Health-aligned malaria control and elimination strategies targeting both human and animal populations. In doing so, the study contributes to national efforts to achieve malaria elimination by 2030, in accordance with Sustainable Development Goal (SDG) 3.3.

## MATERIALS AND METHODS

### Ethical approval and Informed consent

The Health Research Ethics Committee of the Faculty of Public Health, Universitas Sriwijaya, approved the study (ethical approval No: 277/UN9.FKM/TU.KKE/2024). Participation in the study was voluntary, and all analyses were conducted using anonymized participant identification codes to ensure maximum confidentiality.

### Study period and location

The study was conducted from March to July 2024 in two subdistricts, Lawang Kidul and Tanjung Agung, located in Muara Enim District, South Sumatra Province, Indonesia.

### Study design

A cross-sectional study design was employed to estimate the prevalence of *Plasmodium* spp. in humans and cattle within a malaria-endemic region of Indonesia.

### Sampling strategy and blood sample collection

This study employed a stratified random sampling strategy, with villages serving as strata to account for geographic and transmission heterogeneity. Human participants were eligible if aged ≥5 years, had resided in the area for at least six months, and had not taken antimalarial drugs within the 14 days prior to sampling. Domesticated cattle aged ≥6 months were selected proportionally from the same households based on herd size. Sample size estimation was based on one-sided hypothesis testing for population proportions, using different parameters for humans and cattle. For humans, the test used a null proportion (P_o_) of 0.0103 and an anticipated proportion (P_a_) of 0.0412. For cattle, P_o_ was 0.0412 and P_a_ was 0.143. Both calculations used a significance level (α) of 0.05 and a power (1–β) of 80%. These yielded minimum sample size requirements of 117 individuals and 38 cattle, respectively. These values were included in the conventional technique for estimating population proportions:


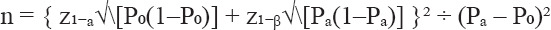


From the 117 surveyed individuals, 41 were purposively selected for PCR-based blood analysis, targeting respondents with malaria history, clinical symptoms, forest exposure, or livestock ownership. For cattle, a complementary prevalence-based estimation (95% confidence level, 5% precision, 10% expected prevalence, plus 10% buffer for non-response) supported a final target of 43 blood samples, all of which were collected. This dual-estimation framework ensured methodological rigor while maintaining consistency between behavioral data and parasitological findings across host species.

### DNA extraction and molecular detection

DNA was extracted from the collected blood samples using the DNeasy Blood and Tissue Kit (Qiagen, Hilden, Germany) according to the manufacturer’s instructions to ensure consistent and reproducible results. Detection of *Plasmodium* spp. was conducted using nested PCR based on genus-specific primers described by Singh *et al*. [8]. The first round of PCR amplification employed the primers rPLU1 (TCA AAG ATT AAG CCA TGC AAG TGA) and rPLU5 (CCT GTT GTT GCC TTA AAC TCC), followed by a second round using primers rPLU3 and rPLU4.

### PCR amplification conditions

PCR amplification was performed using a GoTaq® G2 Green Master Mix (Promega, USA) in a total reaction volume of 25 μL. The reaction mixture consisted of 12 μL of GoTaq Green Master Mix, 6 μL of nuclease-free water (ddH_2_O), 1 μL each of primers rPLU1 and rPLU5, and 5 μL of DNA template. This mixture was used as the template for the first round of nested PCR. subsequently, 2 μL of the first-round PCR product was used as the template for the second-round PCR, which employed primers rPLU3 and rPLU4. Thermocycling was performed under standardized conditions, starting with an initial denaturation at 95°C for 5 min, followed by 35 cycles consisting of denaturation at 95°C for 30 s, primer annealing at 55°C for 1 minute, and extension at 72°C for 1 min. The amplification concluded with a final extension step at 72°C for 4 min.

### Gel electrophoresis

The amplified PCR products were analyzed thr-ough 1.5% agarose gel electrophoresis. Samples were considered positive for *Plasmodium* spp. if a distinct band at 240 base pairs (bp) was observed. This rigorous methodological framework ensured high accuracy and reproducibility, minimizing contamination and procedural biases, thus reinforcing the validity of the findings and their applicability to malaria control strategies.

### Advantages of molecular techniques

The application of endpoint PCR enabled precise assessment of malaria prevalence across host species, building upon the foundational study by Singh *et al*. [8]. The assay’s capability to detect both genus- and species-level infections enhanced the reliability of the results. Although microscopy remains an important diagnostic tool, its limitations in detecting mixed infections or low parasitemia levels, as previously reported, underscore the superiority of molecular techniques for comprehensive malaria surveillance.

## RESULTS

The study demonstrated a notable disparity in infection rates between humans and cattle. No infections were detected among the 41 human samples, resulting in an infection rate of 0%. Conversely, one infection was identified among the 43 cattle samples, corresponding to an infection rate of approximately 2.33%. The relative risk (RR) for humans was 0.0%, indicating no observed risk of *Plasmodium* spp. infection, whereas cattle exhibited an RR of 2.33%, reflecting a low but existing risk. These results confirm the effectiveness of current public health measures in preventing zoonotic malaria transmission among humans, while highlighting the potential role of cattle as reservoirs for *Plasmodium* parasites. The observed difference in infection rates highlights the necessity for integrated surveillance strategies that combine human and veterinary health efforts to effectively monitor and mitigate zoonotic malaria risks.

### Study participants

The prevalence of *Plasmodium* spp. in blood sam-ples from humans and cattle was investigated using the endpoint PCR method at the Biomolecular and Genomic Laboratory, Central Environmental Health Laboratory, under the Indonesian Ministry of Health. A total of 41 human blood samples and 43 cattle blood samples were successfully collected and analyzed. The cattle sampled had an average age of approximately 3 years and varied histories of exposure to mosquitoes. These demographic and environmental factors were considered to better understand the biological and ecological risks associated with *Plasmodium* spp. infection.

### Outcome data

#### Human blood samples

All 41 human blood samples tested negative for *Plasmodium* spp., indicating a 0% infection rate (95% confidence interval [CI]: 0.0%–8.5%) (Table 1). These results suggest that there was no active malaria transmission among the human population in the surveyed region at the time of the study.

**Table 1:** Summary of PCR results for human samples.

No.	LIMS code	Sample type	Results
1	2024A120002	Whole blood human	Negative
2	2024A120003	Whole blood human	Negative
3	2024A120004	Whole blood human	Negative
4	2024A120005	Whole blood human	Negative
5	2024A120006	Whole blood human	Negative
6	2024A120007	Whole blood human	Negative
7	2024A120008	Whole blood human	Negative
8	2024A120009	Whole blood human	Negative
9	2024A120010	Whole blood human	Negative
10	2024A120011	Whole blood human	Negative
11	2024A120012	Whole blood human	Negative
12	2024A120013	Whole blood human	Negative
13	2024A120014	Whole blood human	Negative
14	2024A120015	Whole blood human	Negative
15	2024A120016	Whole blood human	Negative
16	2024A120017	Whole blood human	Negative
17	2024A120018	Whole blood human	Negative
18	2024A120019	Whole blood human	Negative
19	2024A120020	Whole blood human	Negative
20	2024A120021	Whole blood human	Negative
21	2024A120022	Whole blood human	Negative
22	2024A120023	Whole blood human	Negative
23	2024A120024	Whole blood human	Negative
24	2024A120025	Whole blood human	Negative
25	2024A120026	Whole blood human	Negative
26	2024A120027	Whole blood human	Negative
27	2024A120028	Whole blood human	Negative
28	2024A120029	Whole blood human	Negative
29	2024A120030	Whole blood human	Negative
30	2024A120031	Whole blood human	Negative
31	2024A120032	Whole blood human	Negative
32	2024A120033	Whole blood human	Negative
33	2024A120034	Whole blood human	Negative
34	2024A120035	Whole blood human	Negative
35	2024A120036	Whole blood human	Negative
36	2024A120037	Whole blood human	Negative
37	2024A120038	Whole blood human	Negative
38	2024A120039	Whole blood human	Negative
39	2024A120040	Whole blood human	Negative
40	2024A120041	Whole blood human	Negative
41	2024A120042	Whole blood human	Negative

PCR=Polymerase chain reaction, *LIMS=Laboratory Information Management System – internal sample tracking ID used by the Environmental Health Laboratory, Ministry of Health, Indonesia. Recommended by Singh *et al.* [8] and WHO [12].

Table 1 presents a summary of the PCR results for human samples. The endpoint PCR results confirmed that all blood samples were negative for *Plasmodium* spp. infection. The results were recorded on August 28, 2024, and validated by the Head of the Environmental Health Laboratory.

#### Livestock PCR test results

In contrast, the analysis of cattle blood samples revealed that one out of 43 samples tested positive for *Plasmodium* spp., corresponding to an infection rate of approximately 2.33% (95% CI: 0.06%–12.0%) (Table 2). The positive cattle sample (designated S12) displayed a distinct band at 240 bp during electrophoresis, confirming *Plasmodium* infection. This finding highlights the significant disparity in infection rates between humans and cattle, emphasizing the importance of continued surveillance to monitor potential zoonotic reservoirs. Although all human samples tested negative, the presence of *Plasmodium* spp. in cattle, even at low prevalence, necessitates sustained zoonotic surveillance efforts.

**Table 2:** Summary of PCR results for livestock samples.

No.	LIMS code	Sample type	Results
1	2024A120044	Whole blood	Negative
2	2024A120045	Whole blood	Negative
3	2024A120046	Whole blood	Negative
4	2024A120047	Whole blood	Negative
5	2024A120048	Whole blood	Negative
6	2024A120049	Whole blood	Negative
7	2024A120050	Whole Blood	Negative
8	2024A120051	Whole blood	Negative
9	2024A120052	Whole blood	Negative
10	2024A120053	Whole blood	Negative
11	2024A120054	Whole blood	Negative
12	2024A120055	Whole blood	Positive
13	2024A120056	Whole blood	Negative
14	2024A120057	Whole blood	Negative
15	2024A120058	Whole blood	Negative
16	2024A120059	Whole blood	Negative
17	2024A120060	Whole blood	Negative
18	2024A120061	Whole blood	Negative
19	2024A120062	Whole blood	Negative
20	2024A120063	Whole blood	Negative
21	2024A120064	Whole blood	Negative
22	2024A120065	Whole blood	Negative
23	2024A120066	Whole blood	Negative
24	2024A120067	Whole blood	Negative
25	2024A120068	Whole blood	Negative
26	2024A120069	Whole blood	Negative
27	2024A120070	Whole blood	Negative
28	2024A120071	Whole blood	Negative
29	2024A120072	Whole blood	Negative
30	2024A120073	Whole blood	Negative
31	2024A120074	Whole blood	Negative
32	2024A120075	Whole blood	Negative
33	2024A120076	Whole blood	Negative
34	2024A120077	Whole blood	Negative
35	2024A120078	Whole blood	Negative
36	2024A120079	Whole blood	Negative
37	2024A120080	Whole blood	Negative
38	2024A120081	Whole blood	Negative
39	2024A120082	Whole blood	Negative
40	2024A120083	Whole blood	Negative
41	2024A120084	Whole blood	Negative
42	2024A120085	Whole blood	Negative
43	2024A120086	Whole blood	Negative

PCR=Polymerase chain reaction, *LIMS=Laboratory Information Management System – internal sample tracking ID used by the Environmental Health Laboratory, Ministry of Health, Indonesia. Recommended by Singh *et al.* [8] and WHO [12].

Table 2 presents a summary of the PCR results for livestock samples. The PCR analysis confirmed one positive case of *Plasmodium* spp. infection among cattle, yielding a prevalence rate of 2.33%. This result warrants further investigation to clarify transmission dynamics and risk factors associated with livestock in malaria-endemic areas.

### Positive livestock sample analysis

**Figure 1:**
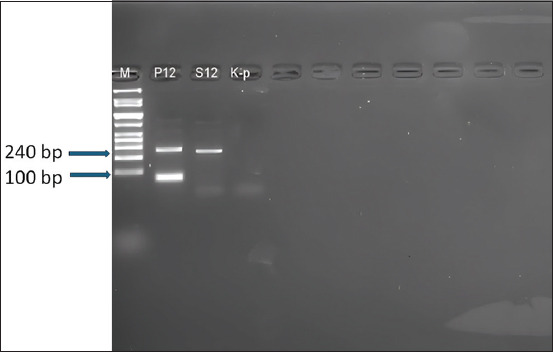
Agarose gel electrophoresis showing the endpoint polymerase chain reaction amplification of *Plasmodium* spp. The DNA band at 240 base pairs in lane S12 indicates a positive cattle sample. Lane P12 is the positive control (*Plasmodium falciparum* DNA); lane K-p is the negative control (nuclease-free water). Universal rPLU primers were used to detect genus-level *Plasmodium* DNA.

The positive cattle sample (S12) was confirmed using endpoint PCR, producing a band at 240 bp, consistent with *Plasmodium* spp. infection (Figure 1). Electrophoresis results depicted a distinct band for sample S12, whereas the positive control (P12) and the negative control (K-p) validated the assay’s accuracy. The universal primers (rPLU1-rPLU5 and rPLU3-rPLU4) used in this study target conserved regions of *Plasmodium* DNA and have been previously validated for genus-level detection. However, the current evidence is insufficient to confirm whether these primers effectively detect cattle-specific species such as *Plasmodium bubalis*. Consequently, while *Plasmodium* DNA was detected, the specific parasite species remains unidentified.

### Combined analysis of human and livestock samples

**Figure 2:**
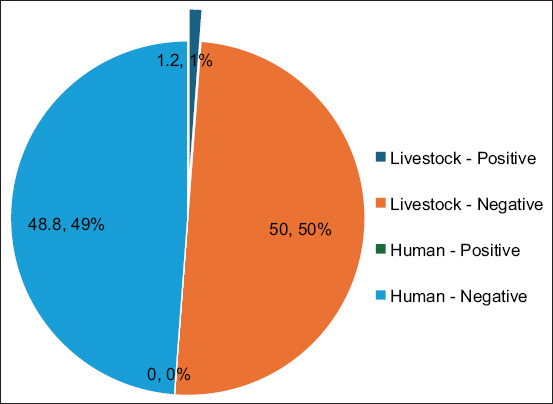
Combined endpoint polymerase chain reaction results for humans and cattle. All 41 human samples were negative. A single cattle sample (S12) was positive for *Plasmodium* spp. The detection was confirmed by a 240-base pairs band.

The combined PCR results for humans and cattle are illustrated in Figure 2, demonstrating a predominance of negative results across both groups. All 41 human samples tested negative for *Plasmodium* spp., reaffirming the absence of active human malaria transmission. Conversely, one of the 43 cattle samples was positive, indicating a low (2.33%) but notable infection rate in the livestock population. Although no human cases were detected, the presence of *Plasmodium* spp. in cattle highlights the potential risk posed by animal reservoirs and underscores the necessity for ongoing integrated zoonotic surveillance. The discrepancy in infection rates between species and the potential reservoir role of cattle is clearly depicted in the combined analysis.

## DISCUSSION

The results highlight a notable disparity in the prevalence of malaria between humans and cattle within the study area. The absence of *Plasmodium* infection among humans suggests that current malaria control measures are effective. However, the detection of *Plasmodium* spp. in cattle raises concerns regarding potential zoonotic reservoirs that could facilitate future malaria transmission if not adequately managed. The absence of *Plasmodium* spp. infections in human samples suggest that public health interventions have effectively mitigated zoonotic malaria risks. Nevertheless, the detection of *Plasmodium* spp. in cattle suggests a potential reservoir for the parasite. The detection of *Plasmodium* spp. in cattle underscores the necessity for continuous monitoring and the adoption of integrated approaches for zoonotic disease prevention. The findings of this study emphasize the need for ongoing surveillance of *Plasmodium* spp. in cattle populations to prevent future outbreaks. Researchers and public health authorities should collaborate to develop integrated surveillance strategies that monitor and control the transmission of zoonotic and reservoir host diseases. Future research should prioritize the accurate identification of *Plasmodium* species in cattle using species-specific primers and advanced molecular techniques, providing more precise insights into the parasite’s taxonomy and potential zoonotic threats. Furthermore, studies should explore transmission dynamics, with particular emphasis on cross-species transmission, to elucidate the role of cattle in broader malaria ecology. Strengthening public health strategies through integrated surveillance systems that encompass both human and veterinary health measures is vital for effectively mitigating zoonotic risks and ensuring a comprehensive approach to monitoring and controlling malaria outbreaks.

These findings align with those of previous studies, which emphasize the role of animals in malaria transmission dynamics. For instance, Hasyim *et al*. [4] found that keeping cattle, particularly medium-sized animals, inside homes was associated with an increased risk of malaria in rural Indonesia. Participants who raised cattle indoors were nearly 3 times more likely to contract malaria than those who did not keep cattle. Although our study did not detect *Plasmodium* infection in humans, the presence of the parasite in cattle suggests that animals could serve as reservoirs, potentially facilitating transmission under certain conditions.

### Regional parallels and ecological concerns

Similarly, zoonotic malaria has emerged as a significant public health concern in Southeast Asia. In Malaysia, *P. knowlesi*, a parasite naturally found in macaques, has become a significant cause of human malaria, particularly in Sabah and Sarawak [1, 3]. The rise in *P. knowles*i infections has been linked to factors such as deforestation, changes in vector behavior, and increased human exposure to forested areas where vectors and reservoir hosts co-exist [3]. While our study did not identify the specific *Plasmodium* species in the positive cattle sample, universal primers detecting the genus level indicated that a potential cross-species transmission could have occurred, with potential implications for human health. Understanding the interactions among vectors, hosts, and the environment is critical for effective malaria control. Mair *et al*. [9] emphasized the importance of studying parasite immunity in the context of genetic and environmental variations, suggesting that comprehensive approaches are needed to address complex disease dynamics. Our detection of *Plasmodium* spp. in cattle aligns with this perspective, underscoring the need for integrated studies that consider multiple variables influencing malaria transmission.

### Role of public health interventions

The absence of *Plasmodium* infection in human samples likely reflects the effectiveness of public health interventions, including the widespread use of insecticide-treated bed nets, indoor residual spraying, and enhanced access to diagnosis and treatment. These measures may have successfully reduced the number of human malaria cases, as indicated by the 0% infection rate among our participants. However, infected cattle suggest that vectors may still acquire the parasite from animals, posing a potential risk of zoonotic transmission. Alternative explanations for the presence of *Plasmodium* spp. in cattle include mechanical transmission through veterinary equipment or biting flies, as well as infection by non-zoonotic species, such as *P. bubalis*. These possibilities highlight the importance of species-level identification for future surveillance efforts.

### One Health perspective and environmental links

This situation reinforces the importance of adopt-ing the One Health approach, which advocates integrat- ed efforts across human, animal, and environmental health domains. Changes in environmental and sociocultural factors can affect the distribution and prevalence of parasitic zoonoses [1]. Incorporating cattle management into malaria control programs could help mitigate the risks posed by animal reservoirs. For example, Kaltsum *et al*. [6] reported the detection of *Plasmodium* spp. parasites in cattle in Muara Enim Regency, supporting the notion that cattle can harbor malaria parasites and potentially influence transmission dynamics.

### Vector identification and control strategies

Understanding the interactions between vectors, hosts, and the environment is crucial for malaria control. Tananchai *et al*. [10] emphasized the importance of precise species identification of *Anopheles* mosquitoes, as species complexes can significantly influence vector control strategies. In Thailand, accurate identification of malaria vectors using molecular methods has enabled targeted control operations and improved our understanding of vector behavior and distribution. Applying similar approaches in Indonesia could enhance our understanding of local vector species, feeding behaviors, and roles in transmitting *Plasmodium* spp. between cattle and humans.

### Innovative control measures

Moreover, innovative vector control tools are necessary to address residual malaria transmission, particularly in areas where conventional interventions are limited. Ruiz-Castillo *et al*. [11] proposed insecticide-treated cattle (ITL) as a potential novel strategy to reduce malaria by targeting zoophilic mosquitoes that feed on animals. Implementing ITL within a One Health framework can improve cattle productivity and minimize human malaria risk. Although their study focused on Africa, the concept could be applied to Indonesia, where cattle play a significant role in rural communities and the dynamics of malaria transmission.

### Strategic integration and surveillance

Our findings underscore the need for integrated malaria control strategies that consider the human-cattle-vector interface. Utilizing cattle management in malaria control programs could mitigate the risk to animal reservoirs. This approach aligns with the One Health concept, advocating for collaborative efforts across the human, animal, and environmental health sectors [5]. These findings reinforce the need for integrated surveillance systems under the One Health framework that address human, cattle, and environmental factors to prevent possible zoonotic spillover from animal reservoirs.

### Alignment with global elimination goals

The findings of this study provide significant insights into the potential role of cattle as reservoirs for *Plasmodium* spp., emphasizing the importance of integrated approaches to malaria control in endemic regions of Indonesia. Despite the absence of *Plasmodium* spp. in human samples, detecting *Plasmodium* DNA in cattle highlights the potential zoonotic transmission risks, which is critical for the malaria elimination agenda aligned with SDG 3.3 [12].

### Cross-validation with other studies

The detection of *Plasmodium* spp. in cattle in this study supports similar observations made by Munirah *et al*. [7], who identified *P. falciparum* and *P. vivax* in West Sumba and Fakfak cattle. Our study also confirmed the presence of *Plasmodium* spp. in cattle. However, unlike Munirah *et al*. [7], our study found no human infections, indicating that the presence of a reservoir does not always correspond with active zoonotic transmission. In addition, the study by Singh *et al*. [8] established the utility of genus- and species-specific nested PCR assays, which have proven sensitive and effective for identifying malaria parasites in remote or field-based studies. The molecular diagnostic methods used in the current study enabled the precise detection of *Plasmodium* spp., even at low prevalence levels, such as 2.33% in cattle samples. These observations corroborate that cattle can act as reservoirs, complicating malaria elimination efforts. Similarly, Lempang *et al*. [13] highlighted the emerging challenge of zoonotic malaria in Indonesia, emphasizing the connection between human interactions with non-human primates and environmental changes, such as deforestation, which facilitates the spillover of pathogens. Although this study focused on cattle, the implications for zoonotic transmission parallel those observed in primate malaria.

### Diagnostic strength and multidisciplinary need

The results underscore the importance of adopting a One Health approach to malaria control that integrates human, animal, and environmental health strategies. As highlighted by Tek *et al*. [14], advancements in molecular diagnostic tools, such as endpoint PCR, enhance the ability to detect *Plasmodium* spp. and guide surveillance efforts. These findings underscore the need for targeted interventions to mitigate malaria transmission risks, including the implementation of enhanced cattle management practices and public health awareness campaigns. Moreover, building on the work of Lempang *et al*. [13], this study reinforces the need for multidisciplinary approaches to address zoonotic malaria through environmental conservation, disease management, and vector control.

### Limitations and future research

The detection of *Plasmodium* spp. in cattle, although at a low prevalence (2.33%), suggests the potential for sporadic zoonotic transmission. These findings align with the hypothesis that animal reservoirs can sustain malaria parasites in the absence of human hosts, as posited by Munirah *et al*. [7]. Therefore, public health initiatives should prioritize cattle as a focus for malaria surveillance and control. Interventions such as routine blood testing in cattle, vector control measures around farms, and public education campaigns to reduce human–cattle interactions in endemic areas are crucial to achieving the goal of eliminating malaria by 2030. This study had a limited sample size, particularly of the cattle population, which may restrict the generalizability of the findings.

Furthermore, although *Plasmodium* DNA was detected in one cattle sample, the study did not identify the specific *Plasmodium* species, necessitating further research. Future studies should explore the molecular characterization of *Plasmodium* spp. in larger, more diverse animal populations and assess the role of environmental factors in sustaining these reservoirs, building on the frameworks proposed by Lempang *et al*. [13] and Tek *et al*. [14]. These findings contribute to the growing body of evidence on zoonotic malaria, reinforcing the need for integrated control strategies. This study emphasizes the significance of cattle surveillance in identifying potential reservoirs and reducing the risk of malaria resurgence. These findings are further supported by a recent narrative review by Hasyim [15], which examines malaria determinants in low-endemic regions such as South Sumatra. By addressing these challenges through One Health initiatives, Indonesia can strengthen its commitment to global malaria elimination goals. As emphasized by Hasyim [15], implementing continuous surveillance, fostering community engagement, and designing targeted, evidence-based interventions will be essential to ensuring effective and sustainable malaria control in an evolving epidemiological landscape.

## CONCLUSION

This study provides valuable insights into the complex relationship between cattle and malaria transmission in Indonesia, where zoonotic diseases remain a significant public health concern. While no *Plasmodium* spp. Infections were detected in human samples, reflecting the effectiveness of current malaria control interventions. The identification of *Plasmodium* DNA in 2.33% of cattle samples underscores the potential role of livestock as reservoirs capable of sustaining or reintroducing malaria under conducive ecological conditions. These findings reinforce the importance of integrating animal health surveillance into malaria elimination strategies under the One Health framework.

Although control efforts, such as insecticide-treated bed nets, indoor residual spraying, and improved access to diagnostics, have significantly reduced human malaria incidence, the presence of *Plasmodium* in cattle raises concerns about zoonotic spillover, especially in rural communities with close human–animal interactions. Mechanical transmission through veterinary practices, infection by non-zoonotic species such as *P. bubalis*, and exposure to zoophilic mosquitoes further complicate the transmission landscape. The absence of species-level identification in this study, due to the use of genus-specific primers, limits conclusions regarding zoonotic potential and highlights the need for advanced molecular diagnostics, including species-specific PCR and sequencing.

Several limitations must be acknowledged. The small sample size, cross-sectional design, and lack of vector data, including *Anopheles* species identification and host-feeding preferences, restrict the generaliz-ability and ecological interpretation of the findings. Future research should adopt longitudinal designs with broader geographical coverage and incorporate entomological, behavioral, and environmental assessments to gain a deeper understanding of transmission dynamics. In addition, socioeconomic determinants such as poverty, limited health literacy, and dependence on cattle for livelihood must be addressed through education, community engagement, and integrated policy interventions.

To mitigate the risks of zoonotic malaria, national malaria control programs should institutionalize coordinated, cross-sectoral strategies. These include routine livestock screening, targeted vector control at the human–animal interface, capacity building for species-level parasite identification, and public awareness campaigns on the risks associated with close human-cattle proximity. A unified surveillance model involving the Ministry of Health, the Ministry of Agriculture, and local stakeholders is essential to operationalize a One Health-based malaria elimination roadmap aligned with SDG 3.3. By strengthening diagnostic capacity, expanding surveillance coverage, and implementing integrated vector management, Indonesia can enhance its resilience against zoonotic malaria and sustain momentum toward national malaria elimination by 2030.

## DATA AVAILABILITY

The datasets are not publicly available but can be obtained from the corresponding author upon reasonable request.

## AUTHORS’ CONTRIBUTIONS

HH: Conceptualization, data curation, project administration, supervision, formal analysis, validation, manuscript drafting, and visualization. BJ: Language correction, Supervision, manuscript editing and revision, literature review, and coordination of institutional collaboration. YY: Conceptualization, data curation, formal analysis, methodology development, validation, and manuscript drafting. FEM: Conceptualization, data curation, investigation, visualization, and sample processing. DD: Conceptualization, investigation, and sample collection coordination. LP: Conceptualization, investigation, and logistics coordination for field sampling. LH: Conceptualization, investigation, and technical support during molecular analysis. ES: Investigation, laboratory supervision, and quality assurance of data collection. MM: Resource provision, supervision of field activities, and manuscript drafting. IAL: Methodology development, software support for data handling, and validation. FHT: Field investigation, assistance with blood sample collection, and documentation of field data. SH: Supervision of laboratory methods, quality control of PCR assays, and manuscript drafting. RY: Supervision, coordination of field data collection, critical revision of the manuscript, and compliance with ethical protocols. All authors have read and approved the final manuscript.
